# Sense of Coherence in Nurses: A Systematic Review

**DOI:** 10.3390/ijerph17061861

**Published:** 2020-03-13

**Authors:** Giuseppe Michele Masanotti, Silvia Paolucci, Elia Abbafati, Claudio Serratore, Michela Caricato

**Affiliations:** 1 Director of Experimental Centre of Research for Health Promotion and Health Education (CeSPES), 06129 Perugia, Italy; 2 School of Specialization in Hygiene and Preventive Medicine, University of Perugia, 06129 Perugia, Italy; silvia.paolucci01@gmail.com (S.P.); elia.abbafati@gmail.com (E.A.); claudio.serratore@gmail.com (C.S.); michelacaricato@gmail.com (M.C.)

**Keywords:** nurses, salutogenesis, Antonovsky

## Abstract

*Background*: Nurses experience high levels of distress due to the nature of their work and workplaces; Antonovsky’s salutogenic theory shows that individual and work-related factors can influence human health. The aim of this paper is to analyze the possible correlations with different work-related and individual variables, which influence or are influenced by Sense of Coherence (SOC) and verify the possible use of SOC scales to prevent negative health determinants in workplaces. *Methods*: Electronic databases were searched with selected studies compared for sample, sample size, study design and basic results. Cross-sectional studies were reviewed for correlations between individual physical and mental health, distress, burnout, job satisfaction and SOC, with intervention studies used to assess the possible impact of training on nurses’ SOC. *Results*: The review found several correlations between SOC and different work-related variables; but also with several individual characteristics. *Conclusion*: The review found that SOC was predictor of depressive state, burnout, job dissatisfaction among female nurses; therefore, SOC could be a health promoting resource.

## 1. Introduction

Nurses are in the front line in the psychologically demanding everyday-care of patients, which can often undermine their emotional balance, influencing both their physical and mental wellbeing [1]. Moreover formal caregivers are frequently burdened with an excessive workload, high working pressure and demands, spending more time at work than on other dimensions of their lives [2,3]. All together these factors may contribute to the creation of a stressful working environment, which requires great coping abilities.

According to the salutogenic theory [4], one of the most critical determinants of the capacity to cope successfully with distress is the Sense of Coherence (SOC), which shapes the individual experience of a stressful event and allows it to be perceived as meaningful, manageable and comprehensible. This can be achieved by mobilizing efficiently the so-called GRRs (generalized resistance resources), which include biological, material and psychosocial factors, triggering a virtuous cycle and in turn strengthening the personal SOC [5]. Similarly, the Conservation of Resources theory (COR) states that the stress can result from circumstances involving loss of valued resources, and that the desire to preserve the individual resources is the basis of the coping ability [6].

Transposing Antonovsky’s theory on the working context, the SOC can be modified, in a positive or negative way, by the nature of the current working environment. This re-adaptation explains how “job resources” are an integral part of the GRRs and participate in the modeling of the worker’s SOC, which consequently influences how the so-called job demands (hours and pattern of work, workload, relations among the colleagues and every organizational aspects of a job that require continuous physical and/or psychological effort) are perceived, appraised, faced and overcome [5].

The SOC scale, named initially by Antonovsky’s “Orientation to Life Questionnaire”, investigating the three dimensions of SOC (Meaningfulness, Manageability and Comprehensibility) is available, to date, in two versions: the original form of 29 items (SOC-29) and the shorter version of 13 items (SOC-13). Each item is scored on a 7-point Likert scale, ranging 29–203 and 13–91, respectively, with higher score corresponding to a more developed SOC.

The aim of this paper is to analyze the possible correlations with different work-related and individual variables, which influence or are influenced by SOC and verify the possible use of SOC-29 or SOC-13 to prevent negative health determinants in workplaces.

## 2. Materials and Methods

### 2.1. Research Methods

A systematic search was conducted up to January 2019 on major healthcare databases: PubMed, Web of Science and Scopus. The following terms were included: sense of coherence, nurse, nurses, nursing, nursing staff, formal caregiver, formal caregivers; no additional filters used. Additional articles were retrieved from the consultation of relevant authors and paper’s bibliography.

### 2.2. Articles Selection

Two independent reviewers selected the studies according to the following inclusion criteria: (1) original articles, and (2) administration of the SOC questionnaire to a sample of formal nurses.

Exclusion criteria applied were: (1) SOC questionnaire not administered, (2) language other than English, (3) sample different from working nurses (nurse teachers, unemployed nurses), (4) impossibility to retrieve a specific SOC value for the nurse sample, (5) absence of both mean SOC value and type of SOC questionnaire, and (6) use of other SOC questionnaire other than SOC-29 or SOC-13. Disagreements on article selection were resolved by consensus.

### 2.3. Data Extraction and Synthesis

Extraction of paper’s data was independently performed by the reviewers through a pre-set table and consensus was reached, upon common revision, for each item inserted therein.

Selected papers were subsequently divided into three categories, based on whether the field of investigation of the Sense of Coherence was work-related or within the individual’s sphere; articles assessing SOC variation upon interventions were categorized separately. The categories were named “Work-Related Variables”, “Individual Variables” and “Interventions”.

## 3. Results

**Figure 1:**
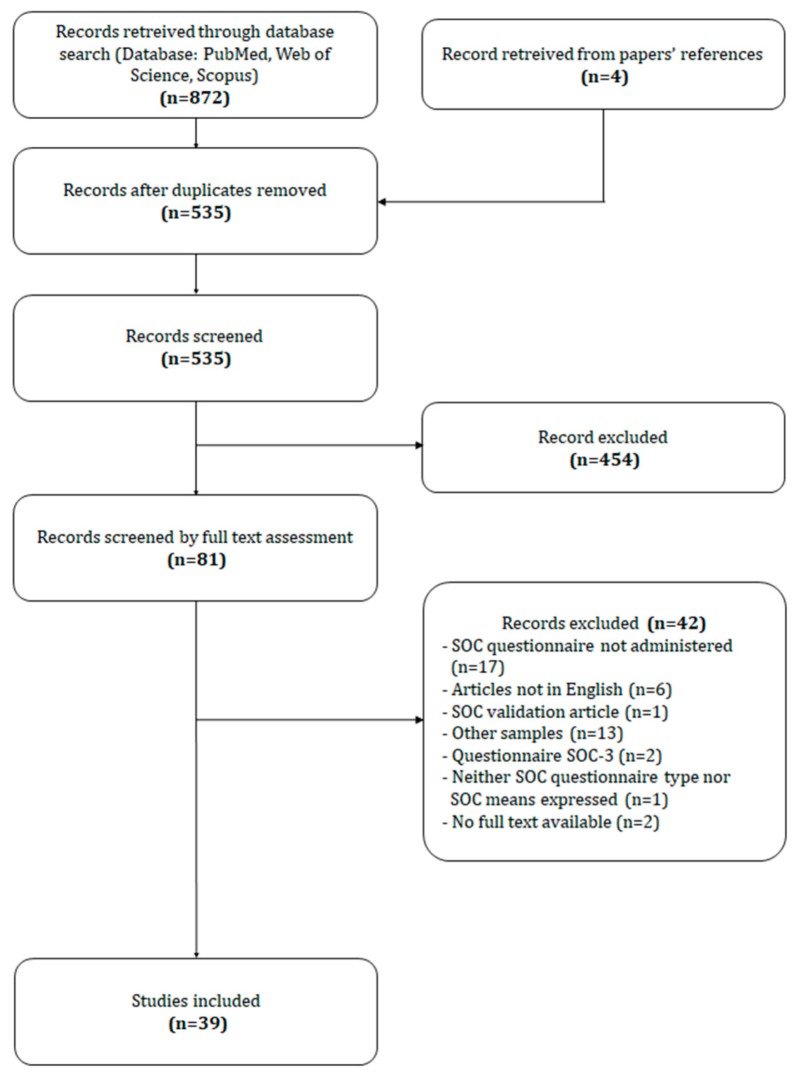
Flowchart of the searching and screening of literatures.

A total of 876 papers were obtained. After duplicates removal, 535 records were screened initially by title and abstract and then by full text assessment. This process led to the exclusion of *n* = 454 and *n* = 42 articles respectively, yielding a total of 39 records included in the present review (Figure 1). 

**Figure 2:**
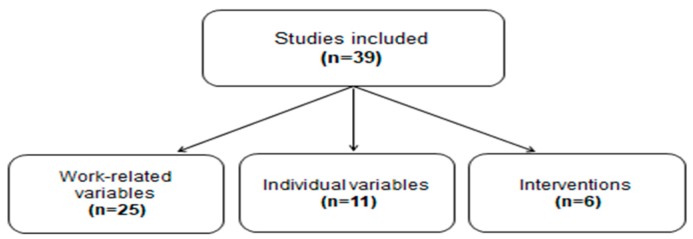
Flowchart of the allocation into the three categories: “Work-related Variables”, “Individual Variables”, “Interventions”.

Data extraction from the included studies were performed and, according to the variables assessed, they were allocated to the three categories mentioned above: “Work-Related Variables”, “Individual Variables”, “Interventions” (Figure 2). Features of the same article, falling into more than one category were assessed separately. Table 1 summarizes the articles.

### 3.1. Work-Related Variables

#### 3.1.1. Job Characteristics

Debska et al. observed among the nurses highest SOC scores for the Manageability subscale (45.15), followed by the Comprehensibility and Meaningfulness subscales. They showed an inverse correlation between SOC and the dimensions of mental load investigated by the Meister questionnaire, such as Monotony, Unspecific Load and Mental Load [6]. The relationship between SOC and general working experience, position at work and employment characteristics was unclear, while some authors found no correlation [7,8], an inverse correlation between SOC and work experience was found by Debska et al. [6], in contrast Miyata et al. [9] observed a positive correlation. Among nurses there is a wide variety of work schedule such as regular, irregular, flexible, etc. Fusz et al. showed that day-shift workers had higher SOC score than shift workers, and that lower SOC value was found among irregular workers (58.19), compared to flexible work schedule workers [10], while Kikuchi et al. observed an inverse correlation between SOC and shift work, job rank, and overtime hours [11]. Several studies found differences of SOC between different professionals, because there was higher SOC in nurses employed as strategic managers [7], Lindmark et al. likewise showed that clinical coordinators have higher SOC score, compared with all other professions, for example dental hygienists have higher scores for meaningfulness, and dentists have higher scores for manageability, compared with dental nurses [12].

Ando et al. described the relationship between the moral distress for nurses and several job characteristics, such as job satisfaction, SOC and mental health, finding an inverse correlation between Moral Distress Scale for Psychiatric Nurses (MDS-P) and SOC. Inverse correlations were found between subscales of the MDS-P and those of SOC [13]. Positive correlation was observed between SOC and workplace adaptability [14], and job satisfaction [11,14,15]. Moreover, Ida et al. identified SOC as an important factor affecting sickness-absence [14].

Lastly planning effective pain and distress management is a crucial part of the nurses’ profession. Hall-lard et al. found that patient’s age and type of illness seems to influence nurses’ assessments of pain and distress, nurses with high emotional stability and high SOC scores assess pain and distress for acute patients as less intense and assess it more intense for chronic patients [16].

#### 3.1.2. Work-Life Balance

Some authors, investigating Work-Life Balance as the proportions of percentages of time spent at work and private life (50/50 and below, 60/40, 70/30, 80/20 and above), reported significantly higher SOC scores in “50/50 and below” and “60/40” groups, whereas the lowest SOC scores were associated with the “80/20 and above” group [2,17].

As far as the Quality of Life (QoL) is concerned, the “50/50 and below” reported the higher scores for overall QoL and physical health, while the “80/20 and above” group the lowest in the overall QoL, in the physical health domain and in the environment domain. No significant differences among the four groups were observed in terms of social support, job satisfaction, and the psychological and social relationship domains of the QoL [2].

#### 3.1.3. Work Related Trauma

Michael et al. investigated the effect of social and personal resources at work, related to trauma. They observed that nurses who did not report a traumatic event had the strongest SOC. This could be due to some causes, nurses with strong SOC did not perceive an event as traumatic, or in contrast, traumatic events influence the SOC [18].

#### 3.1.4. Social Support

Social support and SOC were found to be significant predictors (*p* < 0.05) for all QoL domains. Indeed, a unit increase in SOC results in a 6–12% increase in the likelihood of having high QoL for all domains, however social support had more influence on nurses’ QoL than their ability to cope with stress [2].

#### 3.1.5. Stress and Burnout

Yam et al. analyzed SOC and perceived stress with a sample of critical care nurses, finding that SOC was a protective factor in relation to stress perceptions arising from the work environment [19].

Höge et al. investigated the possible impact of SOC and negative affectivity on the relationship between work stressors and strain. They found a strong correlation between SOC and negative affectivity [20].

Berg et al. [21] observed that Work-Related Strain Inventory (WRSI), measuring the feeling of psychological strain in occupational setting, and factor involvement of the Satisfaction with Nursing Care and Work (SNCW) scale, negatively related to SOC.

Several studies negatively correlated SOC with overall stress [8,20] and work-related stress [8], especially workload [22]; in these studies, nurses’ overload in the workplace was identified as a critical factor for stress development. Burnout and SOC were found to inversely correlate in several studies [22,23,24,25].

Moreover, burnout subscales were observed to logically relate to SOC. A stronger coping ability is associated with higher scores in personal accomplishment, lower levels of emotional exhaustion, and depersonalization [8,15,26,27]. Workload was considered a major contributing factor for burnout [8].

### 3.2. Individual Variables

#### 3.2.1. Individual Characteristics

Five studies investigated the correlation between SOC and age, with discordant results: two studies [11,28] revealed a positive association between SOC and age of the participants, whereas another three studies [7,8,29] did not find this relationship significant. Nevertheless, in the study conducted by Debska et al., although no significant correlation was found between total SOC and age, an inverse association between age and Manageability subscale was observed [6].

Although one study did not find any correlation with sex of the participants [26], an earlier study by Lewis [8] observed a stronger SOC in women, compared to men.

SOC was associated to marital status in the study conducted by Tselebis et al. [26], whereas the same correlation was not found in other studies [8,9].

Educational background, considered by Kretowicz et al. was found to be positively associated to overall SOC and Meaningfulness [7]. Two studies by Debska et al. and Lewis et al. have not proven the same correlation [6,8].

#### 3.2.2. Individual Physical and Mental Health

The relationship between SOC and nurses’ health was the focus of several studies. Miyata et at. associated positively SOC with good mental health status and good physical health status [9].

Schäfer et al. observed a significant increase from the cut-off value of nurses’ scores in ICD-10-Symptoms Rating (ISR), evaluating general health problems, as well as symptoms burden, depression and eating disorder symptoms. Moreover, when compared to physicians, nurses reported higher ISR and symptoms burden scores, the same was not found for variables such as Resilience, SOC or LOC (Locus of Control). Furthermore, SOC, Resilience, and Internal and External LOC correlated with ISR scores and Post-Traumatic Stress Disorder (PTSD) symptoms, correlating SOC as a significant predictor of mental health problems and of symptom severity [30].

An inverse correlation was found between health risk and SOC, the latter significantly affecting sickness-absences, especially for experienced and expert nurses, for whom it is the only casual factor, among the other investigated variables [14].

Depression and SOC have been found to negatively, and strongly, correlate in several studies [11,26,28,31]. Takeuchi et al. also considered the interaction of SOC and work-family conflicts (WFC) on the degree of nurses’ depression and pointed out the buffering effect of SOC against depression, resulting from WFC [31].

Moreover, an inverse correlation was found between SOC and personal stress [22] and cumulative fatigue [31].

#### 3.2.3. Personality Traits and Characteristics

Van der Colff et al. showed that SOC was correlated positively with different coping strategies, evaluated through the Coping Orientation for Problem Experienced (COPE) questionnaire, namely Approach Coping (seeking emotional/social support) and Turning to Religion; the correlation was inverse for Avoidance and Focus on and ventilation of emotions [15].

Overall a higher SOC score was associated with stronger total coping resources [22], thriving and the use of GRRs [1] and greater self-motivation, measured by the Self-Motivation Inventory (SMI) [32].

SOC was positively related to perceived progress goal as well as perceived control, both related to the perception of characteristic tasks of the job and life activities in which nurses were involved, evaluated upon interruption signals. Such signals were found to have a lower positive affect and higher negative affect in nurses with lower SOC [33].

Few studies concentrated on the relationship between SOC and personality traits. Kikuchi et al. revealed that SOC had a strong correlation with almost all personality traits, the strongest being the one with Neuroticism [11]. Höge et al. underlined the same concordant relationship between SOC and Negative Affectivity [20]. Similarly, SOC was found to correlate to the Karolinska Scale of Personality (KSP): negatively to Impulsiveness, Monotony Avoidance, Detachment, Hostility and Psychasthenia, and positively to Socialization and Empathy [23].

The KSP variable “Somatic Anxiety” was inversely related to SOC [23], but in contrast, no differences in mean SOC between the Anxiety (+) and the Anxiety (−) groups were found by Yoshida et al. [34].

#### 3.2.4. Negative Life Events

In two different studies Hochwälder et al. investigated the association of negative life events on nurses’ SOC [29,35]. There was no strong evidence that negative life events lower SOC in the sample population, but those who experienced a negative life event had initially a weaker SOC, compared to those who did not experience any negative life events [35]. Although there was not a significant correlation between SOC and the number of uncontrolled negative life events, those with high and moderate SOC reported fewer controllable negative life events compared to individuals with low SOC [29].

### 3.3. Intervention Studies

A total of six studies analyzed the effect of an intervention on nurses’ Sense of Coherence. Only two studies observed a significant improvement in the SOC scores [36,37]. In the first study, nurses participated in a modified version of the Mindfulness-Based Stress Reduction (MBSR) program, lasting two weeks. After the intervention, it was observed a significant decrease in GHQ and its subscales (Physical Symptoms, Anxiety/Sleep, Social Activities and Depression), indicating an overall improvement in general health. Furthermore, SOC increase was significant, compared to the control group, as it was the increase in the meaningfulness subscale score, compared to comprehensibility and manageability scores [36].

Sarid et al. investigated the effect of Cognitive-Behavioral Intervention (CBI), comprised of 16 meetings, once a week, on nurses’ SOC. At baseline the two groups did not differ in respect to SOC, perceived stress and mood states [37,38]. At T2 (four months after the beginning, upon completion of the program), nurses of the intervention group scored higher in SOC and vigor scales, whereas reported decreased level of perceived stress and fatigue. Such changes were not reported in the control group.

Nurses in the study conducted by Shimizu participated in an Assertive Training program. Although no significant changes in SOC were reported, the effects of the intervention were appreciable as an improvement in Self-esteem scores in the sample analyzed [39].

Berg 1999 and Pålsson 1996 both investigated the outcomes of systematic clinical supervision strategies on nurses. The two studies did not report significant changes in SOC after the intervention [21,23].

Only one study observed a reduction of mean SOC scores of nurse managers in early years of their supervisory roles, after the participation in a four-month experiential learning-based program [40].

The effect of an IT support project on SOC was considered in one study: no significant within-subject effects for the total SOC scale and meaningfulness subscale was observed both in the group receiving the intervention and the control group. However, IT support improved the perception of psychosocial job satisfaction and the quality of care; in this sense the study showed a significant interaction effect for the family relation factors, close friend relation (LSQ), total SOC scale and meaningfulness subscale [41].

## 4. Discussion

The nursing profession is characterized by taking care of patients and their families, it is a factor increasing the mental and emotional burden, and for this reason nurses’ Sense of Coherence needs to be strong enough to deal with several stressful working experiences. Among others, most of the strain experienced by nurses derives from heavy workload, unsatisfactory work environment and work conditions, deep emotional involvement in others, organizational structure, lack of resources, inter-professional conflicts and professional uncertainty [21].

The majority of nurses spend more time at work than on their private life and report significantly higher SOC scores for those whose percentages were proportionally lower, and the lowest scores were for nurses with higher percentages of time spent on working activities [2,17].

Nurses face moral distress and feel so powerless because of the management policy of institutions [13]. The crucial role of institutions in cooperating in the hospital management is also correlated to a positive perception of safety, which in turn is correlated with absence of burnout and a strong Sense of Coherence [24].

The raising of SOC and organization environment reduces sickness-absence. Improving comprehensibility by enriching professionalism, recovering meaningfulness and manageability through optimizing work-life balance and social support may also raise SOC.

SOC and social support were found to be significant predictors for all QoL domains. Social support had the most relevant influence on nurses’ QoL and is considered as a buffer in the stressful situations of healthcare working environment to help the individuals to cope. Cultivating social support could indeed help the individuals to improve their coping abilities and their general health status [2].

Occupational stress is a major contributing factor to burnout [15]. This correlation is also supported by studies, showing that individuals with high burnout levels are expected to possess poor stress coping abilities, specifically in the manageability dimension of the Sense of Coherence, which was found to be related to emotional exhaustion of burnout [15]. Burnout is defined as a syndrome of emotional exhaustion, depersonalization and decreased sense of self-achievement, unfortunately, occupational burnout affects a considerable proportion of nurses who face daily stress experienced at work [25].

Despite the relative stability of SOC after the third decade, it may be shaped progressively throughout the whole course of someone’s life and the GRRs [42], which are mobilized by the Sense of Coherence, arise from the cultural, social and environmental conditions of living, in addition to idiosyncratic factors [43].

This concept could explain why the analysis of the correlation between SOC and individual characteristics (age, sex, marital status and educational background), taken into account only in few studies, yield discordant results.

An interesting point was explored by Kretowicz et al., who correlated positively SOC and educational background: as SOC is considered to have an educational value and the progress in the academic education could elevate it, it is reasonable to think that this relationship could influence task completion in managerial positions [7].

Furthermore, Antonovsky did not exclude a possible influence of negative life events on SOC, especially for those with low or moderate SOC [42]. Starting from this assumption, Hochwälder et al. in two different studies [29,35] have investigated this relationship: no association was found between negative life events and nurses’ SOC, however those who experienced a negative life event had initially a weaker SOC, compared to those who did not experience any [35]. This result led the authors to consider a low SOC as a vulnerability factor, rather than considering a high SOC as a protective factor. This finding is in accordance with Antonovsky’s assumption that a high SOC could prevent the experience of negative life events, both helping individuals to avoid potential stressors and not allowing them to perceive them invariably as negative [4].

A strong SOC is believed to be related to general well-being [15]. This relationship was confirmed by Miyata et al., who demonstrated in nurses a positive association between SOC and good mental health status and good physical health status [9].

Moreover, nurses working in hospitals reported, compared to the general population, higher burden of general health problems, as well as symptoms of burden, depression and eating disorders symptoms. SOC was found to be the most important predictor for general mental health problems and post-traumatic stress symptoms. SOC could play a crucial role in the development and course of these health issues, by shaping the perception and attitude toward aversive work experiences and stress [30].

The inverse relationship between SOC and health risk and the identification of SOC as a key determinant of sickness absences demonstrates how a poor coping ability, in the presence of powerful stressors, such as advanced career levels, could represent a health risk, due to a decreased ability to cope successfully with the stress. Nevertheless, Ida et al. advanced the possibility that raising SOC and the organization of the environment could produce a positive effect on sickness absences [14].

The inverse correlation between depression and SOC was strong in several studies [26,30,31].

Possession of a strong SOC allowed nurses to better manage occupational stress due to lack of organizational support and job demands, through the choice of appropriate coping strategies [15], to define themselves as thriving, with a positive use of GRRs [1] and to possess a greater self-motivation [32]. Self- motivation was found related to certain specific behavior attitudes (propensity toward physical activity and giving it value in respect to health) and the hypothesis of Langius et al. of a positive relation to SOC was confirmed by their investigation [32].

Only two studies investigated the correlation between anxiety and SOC. Yoshida et al. confronted two groups, divided based on anxiety presence, assessed by an ad hoc questionnaire: no differences were found among the two groups, possibly explained by the initially high SOC possessed by the group at hand [34]. Palsson et al.’s finding indicated that there is an inverse relationship between self-rated pathogenic anxiety and self-rated salutogenic Sense of Coherence [23].

Among the five studies investigating the effects of an intervention on the SOC, only two studies reported significant results. Stress coping strategies improvement, achieved by the MBSR therapy was demonstrated by a significant increase in SOC scores. Moreover, a significant increase in the Meaningfulness subscale of SOC indicated that, through the program, nurses were able to focus their attention on mind and body, allowing them to find meaning in their life and work activities [36].

The effects of CBI were significant in increasing SOC and vigor levels and in decreasing perceived stress and fatigue. CBI aims to raise the personal awareness on possible stress reaction, to learn how to self-talk in anxiety-producing situation, to gain a balance and awareness on perspective stressful events and to facilitate cognitive restructuring of stressful work situations. These intrinsic characteristics of the therapy explain the improvement in nurses’ coping ability and the reduction of negative moods [37,38].

The only study reporting a decrease in mean SOC score investigated the changes produced by an experiential learning-based program. This result was explained by the overload experienced by nurse managers in early years of their supervisory roles when discussing their behavior and stressful situation encountered at work. Furthermore, it has been argued that SOC could possibly increase after an initial decrease, which was not evaluated, due to the short-term follow-up performed [40].

The other intervention studies did no show significant changes on SOC, these results are coherent with the initial description of SOC by Antonovsky, for whom SOC tends to remain stable in adulthood under normal circumstances and can be considered as a moderating factor on negative work environment variables [21]. Moreover, also the well-recognized difficulty in obtaining a significant SOC change in respect to high or low baseline is considered a determining factor of SOC stability in these studies [23].

## 5. Conclusions

SOC provides a solid theoretical basis for examining the organization of work [32].

Therefore it has been proposed that nursing management could focus on building a healthy work environment, which fosters SOC, rather than concentrating on resolving the effects of stress and its management at individual level [33].

Comprehensibility is improved by a clear view on roles and responsibilities and by open communication channels; sense of manageability is strengthened by appropriate workload and availability of resources [8,22]. Participation in decision making and the perspective of a clear career path are factors improving the sense of meaningfulness [8,33].

We found that SOC was a protective factor for depressive state, burnout, job dissatisfaction among female nurses, but there is no clear correlation with factors such as working experience or position at work. In addition, a higher SOC enhances a good mental and physical health status, acting as a health promoting resource, according to Antonovsky’s theory [44,45].

## Figures and Tables

**Table 1:** Included study.

Author	Sample-Size/sex	Sample	Country	Mean Age (SD)	Mean Experience (SD)	SOC Scale	Mean SOC (SD)	Study Design	Other Used Instruments	Sense of Coherence (SOC)-Related Results
Kowitlawkul et al. 2018	1040 nurses 955 females (91.8%) 69 males (6.6%) 16 missing (1.5%)	Registered nurses and enrolled nurses working in inpatient and outpatient departments at a tertiary hospital (in the hospital for ≥6 months)	Singapore	30.6 (8.5)	--	SOC-13	50/50 group: 55.7 (9.5) 60/40 group: 56.0 (9.6) 70/30 group: 54.5 (9.2) 80/20 group: 52.4 (10.8)	Descriptive quantitative study	Work life balance (WLB) Job satisfaction questionnaire Social support questionnaire WHO-Quality of Life (WHO-QOL)-BREF-26 questionnaire	- Division in four groups depending on the proportion of time spent on work and private life (50/50 and below, 60/40, 70/30, 80/20 and above) -“50/50 and below” and “60/40” groups’ SOC scores are higher the other two groups-lowest SOC scores are in the “80/20 and above” group -SOC and social support are significant predictors for all QoL domains -A unit increase in SOC results in a 6–12% increase in likelihood of having high QoL for all domains
Kuraoka et al. 2018	63 nurses 60 females (95.2%) 3 males (4.8%)	Nurse managers in the first 3 years of a supervisory role, working in acute-care hospitals Participation in an experiential learning-based program	Japan	45.2 (3.4)	1.4 (0.6)	SOC-13	Pre-test: 57.17 (10.28) Post-test: 54.97 (10.4)	Quasi-experimental study	Experiential Learning Inventory on the Job (ELI) Ad hoc questionnaire for Knowledge of Experiential Learning Social Support Questionnaire (SSQ) Nurse Managers Competence Inventory (NMCI)	-Mean SOC scores reduction after participation to the experiential learning-based program
Schäfer et al. 2018	27 nurses 44 females (65.4%) 18 males (34.6%)	Nurses working in ICU and anesthesiology unit	Germany	39 (10)	--	SOC-13	43.19 (9.63)	Cross sectional study	Resilience Scale (RS-11) Scale for assessment of internal and external control beliefs (IE-4) ICD 10 symptom rating (ISR) PTSD Checklist (PCL-5)	-No differences in resilience, nor SOC or LOC (locus of control) between physicians and nursing staff -SOC, resilience, and internal and external LOC correlate with ISR score and PTSD symptoms -SOC is a significant predictor of mental health problems and of symptom severity -Resilience, internal and external LOC account for 59% of SOC variance and have a significant indirect effects on symptom severity measures mediated by SOC on symptoms severity measures
Debska et al. 2017	164 nurses (sex unknown)	Nurses working at inpatient chemotherapy wards	Poland	43.07 (7.99)	In chemotherapy ward: 11.77 (7.65)	SOC-29	125.05 (18.30)	Cross-sectional study	Meister Questionnaire	-Highest SOC scores for the Manageability subscale (45.15), lower for Comprehensibility and Meaningfulness subscales (41.18 and 38.73, respectively) - Inverse correlation between SOC and Monotony (r = −0.398, *p* < 0.001), Unspecific Load (r = −0.370, *p* < 0.001), - Mental Load (r = −0.378, *p* < 0.001) and Work experience (r = −0.19, *p* = 0.016) -No correlation between SOC and work-experience -Inverse correlation between age and Manageability level (r = −0.193, *p* = 0.014) -No correlation between overall SOC and age, nor educational level
Fusz et al. 2017	518 nurses 482 females (93.1%) 36 males (6.9%)	Nurses working in eight different hospitals	Hungary	42.44 (9.59)	--	SOC -13	61.76	Cross-sectional study	Ad hoc questionnaire for sense of quality, quality of sleep, frequency of psychosomatic symptoms, and work schedule regularity	-Higher SOC score for day-shift workers (65.84), compared to shift workers (61.02) (t = 2.933; *p* = 0.004) -Lower SOC score for irregular work schedule workers (58.19), compared to flexible work schedule workers (63.17, *p* = 0.04) -Lower scores in self-heath-assessment for irregular work schedule workers, compared to flexible work schedule workers (*p* = 0.019)
Stock et al. 2017	12 nurses 10 females (83.33%) 2 males (16.67%)	Nurses self-described as thriving, experienced (10+ y) or recently retired (<1 y)	Hawaii	55–64 y: 33.33%	10–21 y: 33% >34 y: 67%	SOC-13	73.58	Mixed method, exploratory-descriptive study	Ad hoc questionnaire for thriving view and experience	Possible link between SOC and self-described thriving nurses Nurses have a high SOC and the use of GRRs
Ando et al. 2016	130 nurses 102 females (78.5%) 28 males (21.5%)	Nurses from psychiatric and internal medicine wards at a national hospital	Japan	40–49 y: 35.4%	18.7	SOC-13	53.5 (9.7)	Cross-sectional study	Moral Distress Scale for Psychiatric Nurses (MDS-P) General Heath Questionnaire - 12 (GHQ-12) Job Satisfaction Scale (JS)	Inverse correlation between MDS-P and SOC Inverse correlation between MDS-P and JS “Unethical conduct by caregivers” negatively correlates with Manageability (r = −0.28, *p* < 0.01), Comprehensibility (r =-0.22, *p* < 0.01) and Meaning (r = −0.017, p < 0.05) “Low staffing” negatively correlated with Comprehensibility (r = −0.30, *p* < 0.001) and Manageability (r = −0.22, *p* < 0.01) “Acquiescence to patients’ rights violations” negatively correlates with Manageability (r = −0.31, *p* < 0.01), Comprehensibility (r = −0.28, *p* < 0.01) and Meaning (r = −0.22, *p* < 0,01). “Acquiescence to patients’ rights violations” (standard β = −0,26, *p* < 0.01) and “Meaning” of SOC (standard β = 0.35, *p* < 0.001) influence Job Satisfaction more than other variables.
Lindmark et al. 2016	165 nurses (sex unknown)	Dental nurses working in the Public Dental Service	Sweden	--	--	SOC-13	68.4 (11.0)	Cross-sectional study	Salutogenic Health Indicator Scale (SHIS) Work Experience Measurement Scale (WEMS)	Clinical coordinators have higher SOC score (75.1; SD 10.0), compared with all other professions, including dental nurses Dental hygienists have higher scores for meaningfulness (23.8; SD 3.3), and dentists have higher scores for manageability (22.1; SD 3.6), compared with dental nurses (22.5; SD 3.4 and 20.3; SD 4.0, respectively)
Vifladt et al. 2016	143 nurses 126 females (88.1%) 13 males (9.1%) 4 unknown (2.8%)	Nurses working in ICUs in six hospitals for ≥ 3 months	Norway	41–50 y: 37.4%	≥21 y: 26.1%	SOC-13	72.2 (8.96)	Cross-sectional study	Hospital Survey on Patient Safety Culture (HSOPSC) Bergen Burnout Indicator	Positive safety culture negatively correlates with burnout and positively correlates with SOC (r = −0.451, r = 0.393, respectively, *p* < 0.001). Inverse correlation between SOC and burnout (r = −0.577, *p* < 0.001) Positive correlation between SOC and safety culture at the unit level (β = 0.014, 95%CI = 0.005-0.024, *p* = 0.003) and hospital level (β = 0.017, 95%CI = 0.008–0.026, *p* < 0.001)
Yoshida et al. 2016	430 nurses 416 females (96.7%) 14 males (3.3%)	Public health nurses (after the earthquake, tsunami and Fukushima Daiichi Nuclear Power Station accident following the Great East Japan Earthquake in 2011)	Japan	≥ 50 y: 35.6%	<10 y: 22.8% >10 y: 71.2%	SOC-13	43.0 (7.7)	Cross-sectional study	Ad hoc questionnaire for anxiety	Division in two groups based on anxiety score on a 10-point Likert scale: 1–5 ‘anxiety (−)’ and 6–10 ‘anxiety (+)’ No difference in mean SOC between the anxiety (+) and the anxiety (-) groups Higher ratio of nurses <40 years of age in the anxiety (+) group (*p* < 0.001) Higher ratio of nurses with <10 years of working experience, staff positions and nursing licenses in the anxiety (+) group (*p* < 0.001)
Hochwälder et al. 2015	1012 nurses (all females)	Nurses working at three hospitals and two primary health care settings, ≥ 30 y of age, with no negative life events during the previous year	Sweden	46.6 (8.9)	--	SOC-29	150.21 (20.28) Low SOC (<144) group *n* = 322): 126.35 (13.82) Moderate SOC (144–160) group *n* = 348: 152.20 (4.84) High SOC (>160) group *n* = 342: 170.63 (7.49)	Longitudinal study	Ad hoc questionnaire for Controllable and Uncontrollable Negative Life events	No significant age differences between Low, Moderate and High SOC groups Higher dropout rate in the Low SOC group No significant difference between SOC groups and mean number of uncontrolled negative life events High and Moderate SOC groups have fewer controllable negative life events compared to the Low SOC group (*p* < 0.001)
Kretowicz et al. 2015	310 nurses 301 females (97.1%) 9 males (2.9%)	Nurse managers in selected medical units	Poland	45.7 (6.7)	Mean nursing experience 24.7 (7.2) y Mean managerial experience 8.8 (6.7) y	SOC -29	147.00 (20.47)	Cross-sectional study	--	No correlation between age and SOC Correlation between educational background and SOC (Kruskal-Wallis test 9.04; *p* = 0.029) and Meaningfulness (Kruskal-Wallis test 12.82; *p* = 0.005) No correlation between SOC and general working experience, managerial working experience, position at work and characteristics of employment Higher SOC in nurses employed as strategic managers (U Mann-Wallis test -2.74; *p* = 0.006)
Makabe et al. 2015	1202 nurses 1116 females (93%) 86 males (7%)	Nurses working in three hospitals	Japan	37 (11)	15 (12)	SOC-13	50/50 group (A): 55.7 (9.5) 60/40 group (B): 56.0 (9.6) 70/30 group (C): 54.5 (9.2) 80/20 group (D): 52.4 (10.8)	Cross sectional study	Work-Life Balance (WLB) Work-Life Balance Satisfaction Job Satisfaction Scale WHO Quality of Life (26-item)	Division in four groups depending on the proportions of percentages of time spent on work and private life - WLB status [50/50 and below (A), 60/40 (B), 70/30 (C), 80/20 and above (D)] Group A has a higher SOC score than all other groups (ANCOVA *p* < 0.001) Group D has a lower SOC score than all other groups (ANCOVA *p* < 0.001)
Miyata et al. 2015	1425 nurses 1333 females (94%) 92 males (6%)	Nurse staff (*n* = 1248%–88%) and nurse managers (*n* = 177–12%) working in 10 hospitals	Japan	35.5 (9.9)	12.8 (9.5)	SOC-13	median: 50 (IQR 45–55)	Cross-sectional study	Recognition behavior scale	No significant correlation between SOC and marital status Positive correlation between SOC and good mental health status (OR = 4.07, 95%CI = 2.53–6.53), good physical health status (OR = 1.08, 95% CI = 1.09–2.89), overall work experience (OR = 1.05, 95%CI= 1.04–1.07), with *p* < 0.001, and recognition behaviors by the nurse manager (OR = 1.02 (95% CI = 1.01–1.04), *p* = 0.006
Kikuchi et al. 2014	347 nurses (all females)	Nurses working at a general hospital (intensive care, pediatrics, surgery, oncology, and emergency medicine)	Japan	33.7 (9.2)	--	SOC-13	54.2 (11.9)	Cross-sectional study	Brief Job Stress Questionnaire (BJSQ) K6 short screening questionnaire Ten-item Personality Inventory (TIPI-J)	Age negatively correlates with depressive state (r = −0.18, *p* = 0.00) and positively to SOC (r = 0.27, *p* = 0.00) Inverse correlation between SOC and shift work (r = −0.16, *p* = 0.00), job rank (r = −0.14, *p* = 0.01), and overtime hours (r = −0.33, *p* = 0.00). SOC and depressive state correlate negatively with almost all job stressors and personality traits Inverse correlation between SOC and Neuroticism (r = −0.49, *p* = 0.00). Inverse correlation between SOC and depressive state (r = −0.67, *p* = 0.00 Positive correlation between SOC and job and life satisfaction (r = 0.47, *p* = 0.00)
Kikuchi et al. 2014	348 nurses (all females)	Nurses working at a general hospital (intensive care, pediatrics, surgery, oncology, and emergency medicine)	Japan	34.4 (9.0)	--	SOC-29	124.4 (21.2)	Cross-sectional study	K6 short screening questionnaire Effort-reward imbalance (ERI) scale	SOC (β = −0.46, *p* < 0.001), over-commitment, effort-esteem ratio, and age significantly correlate with the depressive state Age correlates positively with SOC (r = 0.12, *p* < 0.05)
Sarid et al. 2012	36 nurses (sex unknown)	Nurses working both clinical and administrative roles in one major regional hospital, ≥ 5 y of experience Intervention group (*n* = 20): Participation in a Cognitive-Behavioral course Control group (*n* = 16)	Israel	50.6 (10.7)	--	SOC-13	Intervention group T1: 70.83 (7.67) T2: 75.05 (6.7) Control group T1: 72.07 (8.9) T2: 69.61 (7.64)	Pre-post test design, with control	Perceived stress scale (PSS) Profile of Mood states (POMS)	In the intervention group at T2 higher SOC, more vigor, less perceived stress, and less fatigue, compared to T1 At T1 no significant difference in mean SOC scores between the groups At T2 significant difference in mean SOC scores between the two groups F(*p*) = 10.44 (*p* < 0.05)
Ando et al. 2011	28 nurses (sex unknown)	Nurses working in geriatric wards, ≥ 20 y of experience, without severe mental problems Intervention group (*n* = 15): participation in mindfulness based therapy sessions Control Group (*n* = 13)	Japan	--	--	SOC-13	Post-intervention Intervention group: 52 Control group: 53	Pre-post test design, with control	General Health Questionnaire (GHQ) Functional Assessment of Chronic Illness Therapy (FACIT-sp)	- Significant decrease in GHQ after the intervention (improvement in general health) - Significant increase in SOC after the intervention, no change in the control group - Meaningfulness: higher scores and significant increase after intervention: Higher scores and significant increase after intervention, compared to comprehensibility and manageability - No effect on spirituality
Basinska et al. 2011	331 nurses (all females)	Nurses working shifts in three general care hospitals	Poland	34.15 (6.61)	--	SOC-29	136.46 (21.43)	Descriptive quantitative study	Work Related Patterns of Behavior and Experience Questionnaire (AVEM)	Division in work related behavior type groups: type G-healthy, type S-frugal, type A-risk (overburdened), type B-burnout Positive correlation between SOC and healthy type G and the frugal type S (r = 0.50, and r = 0.20 respectively, *p* < 0.001) Inverse correlation between SOC and burnout type B and the overburdened type A (r = −0.57 *p* < 0.001 and r = −0.13 *p* < 0.05 respectively) SOC explains 28% of the variability of type B, 21% of type G and 7% of type S
Hochwälder et al. 2011	1012 nurses (all females)	Nurses working in hospitals and primary health care setting, > 30 y of age Group 0: no negative life events Group 1: ≥1 negative life event in the previous year	Sweden	46.90 (8.85)	46.9 (8.85)	SOC-29	At T1: 150.21 (20.28) At T2: 150.89 (20.38)	Cross sectional study	Ad hoc questionnaire for Negative Life Events	SOC stable from T1 to T2 No strong evidence that negative life events lower SOC No evidence that negative life events lower SOC more in persons with an initially low or moderate SOC that in persons with an initially high SOC. SOC for those who experience a negative life event, is initially weaker, than those who do not experience any negative life events
Sarid et al. 2010	36 nurses (sex unknow)	Nurses working in all hospital wards, > 5 y of experience, both clinical and administrative roles Study group (*n* = 20): participation in a CBI course Control group (*n* = 16)	Israel	50.6 (10.7)	--	SOC-13	--	Pre-post test design	Perceived Stress Scale (PSS) Profile of Mood States (POMS)	No differences in SOC, perceived stress and mood states between the groups at T1 Intervention group at T2: increased levels of SOC and vigor and decreased levels of perceived stress and fatigue Mean changes in SOC were -4,20 (SD = 1.28) for the study group and 2,11 (SD = 1.46) for the control group, F = 10.44, *p* < 0.05
Takeuchi et al. 2010	138 nurses (all females)	Nurses working at three hospitals, who are also mothers and/or wives	Japan	36.2 (8.0)	12.5 (8.3)	SOC-13	56.7 (9.9)	Descriptive quantitative study	Job Content Questionnaire (JCQ, 6 items) Cumulative fatigue symptoms index (CFSI-18) Center for Epidemiologic Studies Depression (CES-D) scale Work-to-Family conflicts scale	Inverse correlation between work-family conflict and SOC (β = −0.233, partial r = −0.340, *p* < 0.01) Inverse correlation between SOC and cumulative fatigue (β = −0.397, *p* < 0.001) and depression (β = −0.517, *p* < 0.001). The interaction of SOC and WFC influences depression (β = 0.214, *p* < 0.05), SOC has a buffering effect on WFC with respect to depression.
Ida et al. 2009	502 nurses (all females)	Nurses working at a major university hospital	Japan	32.4 (9.9)	--	SOC-29	--	Cross-sectional study	Job Content Questionnaire (JCQ-12) Ad hoc questionnaire for medical errors and nurses career levels	Positive correlation between Comprehensibility and professional experience (r = 0.125, *p* < 0.001) Positive correlation between SOC and workplace adaptability (r = 0.335, *p* < 0.01), and job satisfaction (r = 0.280, *p* < 0.01) Inverse correlation between SOC and organization environment (r = −0.611, *p* < 0.01) and health risk (r = −0.364, *p* < 0.01) SOC affects sickness-absence (OR = 0.982, 95%CI = 0.970–0.995)
Van der Colff et al. 2009	818 nurses 791 females (97.4%) 21 males (2.6%) 6 sex unknown	Nurses working in hospital wards, psychiatric wards, community/occupational services and nursing management.	South Africa	40	19	SOC-29	137.95 (20.46)	Cross sectional study	Nursing Stress Inventory (NSI) Coping Orientation for Problem Experienced Questionnaire (COPE) Maslach Burnout Inventory- Human Services Survey (MBIHSS) Utrecht Work Engagement Scale (UWES)	Correlation between SOC and burnout subscores. Emotional exhaustion (−0,49), Depersonalization (−0,47) and Personal accomplishment (0.34) (*p* < 0.05) Positive correlation between SOC and Engagement (0.42) Negative correlation between SOC and Nursing stress inventory (NSI) variables: Lack of organizational support (−0.23), job demands (−0.28) and nursing-specific demands (−0.15) (*p* < 0.05) SOC correlates with coping strategies: Approach coping (0.0), Seeking emotional/social support (0.18) and Turning to religion (0,11), Avoidance (−0.35) and Focus on and ventilation of emotions (−0.29) (p < 0.05)
Hall-Lord et al. 2006	71 nurses (sex not specified)	Nurses participating in an educational program	Sweden	39.2 (8.05)	16.7 (7.35)	SOC-13	--	Cross-sectional study	Ad hoc questionnaire for assessment of pain and distress of patients Five-factor personality inventory (FFPI)	Patients’ age and type of illness seems to influence nurses’ assessments of pain and distress Nurses with high emotional stability and high SOC scores assess pain and distress for acute patients as less intense and assess it more intense for chronic patients
Engström et al. 2005	33 nurses 31 females (94%) 2 males (6%)	Nurses working in a residential home for persons with dementia Experimental group (*n* = 17): participation in IT support project Control group (*n* = 16)	Sweden	41	Experimental group -in nursing care 12 (8) -in dementia care 5 (3) Control group -in nursing care 15 (9) -as district nurse 9 (7)	SOC-13	Intervention group -baseline: 72 (12) -at 6 months: 69 (11) -at 12 months: 75 (9) Control group -baseline: 68 (10) -at 6 months: 69 (9) -at 12 months: 65 (13)	Quasi-experimental non-equivalent groups design	Satisfaction with Work Questionaires (SWQ) Life Satisfaction Questionnaire (LSQ)	Perception of psychosocial job satisfaction and quality of care improve in the experimental group No significant within-subject effect for the total SOC scale and meaningfulness subscale, split on the experimental and control groups Interaction effect for the factors family relation, close friend relation (LSQ), total SOC scale and meaningfulness subscale
Höge et al. 2004	160 nurses (sex not specified)	Nurses working at two hospitals	Germany	--	--	SOC-13	68.1 (10.83)	Cross-sectional study	Work load screening TAA-KH-S Negative Affectivity Scale (NAS) Maslach Burnout Inventory (Emotional exhaustion subcategory) Irritation Strain questionnaire Short Form Health Survey (SF-12)	Inverse correlation between SOC and Negative Affectivity (r = −0.61) Inverse correlation between SOC and overall strain (rs = −0.33) and overall stressors (rs = −0.34)
Shimizu et al. 2004	285 nurses (all females)	Nurses working at a hospital Intervention group (*n* = 62): participation in an assertiveness training Reference group (*n* = 196)	Japan	Intervention group: 44 (7.1) Reference group: 27.8 (5.9)	--	SOC-13	Intervention group T1: 57.4 (10.5) T2: ΔSOC 2.2 (8.3) Comparison group T1: 52.7 (9.4) T2: ΔSOC 1.5 (8.6)	Pre-post test design, with control	Rosemberg’s self-esteem scale (SES)	-No significant difference between the ΔSOC of the intervention group and that of the reference group. -Improvement in SES at six months after the intervention
Cilliers et al. 2003	105 nurses (all females)	Nurses working in large hospitals, ≥ 5 y of experience	South Africa	range: 28-57		SOC-29	141.28 (16.44)	Cross-sectional study	Maslach Burnout Inventory (MBI) Personal Views Survey (HAR) Self-Control Schedule (LR)	Inverse correlation between burnout and salutogenic functioning (SOC, HAR and LR) Inverse correlation between SOC and emotional exhaustion (r = −0.21, *p* < 0.01) and depersonalization (r = −0.25, *p* < 0.01) Positive correlation between SOC and personal accomplishment (r = 0.35, *p* < 0.001)
Yam et al. 2003	29 nurses 28 females (96.5%) 1 male (3.4%)	Nurses working in critical care in public and private hospitals	Hong Kong	32	7.6	SOC-13	--	Cross-sectional study	Critical Care Nursing stress scale (CCNSS) Perceived Stress Scale (PSS)	Inverse correlation between SOC and CCNSS (r = −0.20, *p* = 0.30) Inverse correlation between SOC and PSS (r = −0.64, *p* < 0.001)
Michael et al. 2001	233 nurses 225 females (96.56%) 8 males (3.43%)	Nurses working in operating suites in private and public hospitals	Australia	41	11 years in operating suite	SOC-13	66.75 (9.77)	Mixed method trianglulated study	Ad hoc questionnaire for work-related traumatic events Ad hoc questionnaire for social support	nurses who did not experience a traumatic event had a higher SOC (t(231) = −3.12, *p* < 0.005)
Tselebis et al. 2001	79 nurses 62 females (78.5%) 17 males (21.5%)	Nurses working in general internal medicine, general surgery and respiratory medical wards in a major hospital	Greece	37.7 (5.5)	11.0 (6.3)	SOC-13	63.60 (11.70)	Cross-sectional study	Maslach Burnout Inventory (MBI) Beck’s Depression Inventory (BDI)	No differences in SOC between sexes nor marital status Inverse correlation between SOC and BDI (r = −0.58, *p* < 0.05) Correlation between SOC and MBI categories, negative correlation with sentimental exhaustion (r = −0.55, *p* < 0.05) and depersonalization (r = −0.45, *p* < 0.05), positive correlation with personal achievement (r = 0.44, *p* < 0.05)
Levert et al. 2000	94 nurses 67 females (71.3%) 27 male (28.7%)	Nurses working in psychiatric units	South Africa	39	--	SOC-13	60.61 (12.42)	Cross-sectional study	Maslach Burnout Inventory (MBI) Work Load and Lack of Collegial Support. Role Conflict and Role Ambiguity	Correlation between SOC and MBI’s components: emotional exhaustion r = 0.41 (*p* < 0.0001) and depersonalization r = 0.36 (*p* > 0.001) SOC (t = 4.48; p.OOO) and work load (t = 4.50; p.OOO) together explain the majority of the variance in emotional exhaustion (36.6%) SOC (t = 3.51; p.OOl) and work load (t = 2.61; p.Oll) together explain the majority of the variance in depersonalization (21.3%)
Berg et al., 1999	22 nurses 16 females (72.7%) 6 male (27.3%)	Nurses working in psychiatric ward, during a year of systematic clinical supervision	Sweden	39.7 (7.1)	13.5 (8.3)	SOC-29	T1: 146.6 (21.2) T2: 153.6 (18.3)	Pre-post-test design	Creative Climate questionnaire (CCQ) Work-related strain inventory (WRSI) Satisfaction with nursing care and work (SNCW)	Not significant Improvement in SOC scores at T1 and T2 Inverse correlation between SOC and WRSI (r = −0.48, *p* < 0.05) Inverse correlation between SOC and SNCW, factor involvement (r = −0.46, *p* < 0.05)
Shiu et al., 1998	20 nurses (all females)	Public health nurses (females, ≥ 1 children, promoted to nursing officer or in charge of a center, unit or team) Work perturbed by interruption signals	Hong Kong	39	--	SOC-29	135.75 (12.27)	Cross-sectional study	Experience sampling diary (ESD)	Positive correlation between SOC and perceived goal progress and perceived control Low SOC group (scores <136) has a lower positive affect [t(323)= - 6.79, p < 0.001] and higher negative affect [t(321.98)= 2.88, p < 0.005], than the high SOC group (≥136) in response to interruption signals.
Pålsson et al., 1996	33 nurses (all females)	District nurses working in 10 primary health care districts Supervisory group (n=21): participation in a training program Comparison group (*n* = 12)	Sweden	Supervisory group: 49.0 (7.1) Comparison group: 46.3 (8.3)	Supervisory group -in nursing care 24.0 (8.2) -as district nurse 16.1 (6.5) Comparison group -in nursing care 21.8 (6.9) -as district nurse 14.0 (7.3)	SOC-29	Supervisory group T1: 148 (17.5) T2: 151 (16.6) Comparison group T1: 154 (13.6) T2: 153 (17.3)	Pre-post test design, with control	Karolinska Scales of Personality (KSP) Burnout Measure Empathy Construct Rating Scale (ECRS)	Burnout, empathy and SOC scores are concordant in respect to the KSP variables Inverse correlation between SOC and KSP variables: somatic anxiety (r = −0.44, *p* < 0.05), impulsiveness (r = −0.40, *p* < 0.05), monotony avoidance (r = −0.42, *p* < 0.05), detachment (r = −0.44, *p* < 0.05), hostility (r = −0.37, *p* < 0.05) and psychasthenia (r = −0.40, p < 0.05) Positive correlation between SOC and KSP variable, socialization (r = 0.44, *p* < 0.01) Inverse correlation between SOC and burnout (r = −0.69, *p* < 0.001) Positive correlation between SOC and empathy (r = 0.76, *p* < 0.001) No significant change in burnout, empathy and SOC over time within the groups nor between the groups at T1 or at T2
Lewis 1994	49 nurses (all female)	Nurses working in dialyses units	USA	39.6	--	SOC-29	148.7	Cross-sectional study	Perceived Stress Scale Nursing Stress Scale Coping Resources Inventory Myers-Briggs Type Indicator (MBTI) Maslach burnout inventory (MBI)	Inverse correlation between SOC and personal stress (r = −0.715) Inverse correlation between SOC and work stress (r = −0.436) Inverse correlation between SOC and burnout (r = −0.395) Positive correlation between SOC and total coping resources (r = 0.667)
Langius et al., 1992	57 nurses (all females)	Nurses participating in an in-house training program (total 5 groups) Sample 1 (*n* = 35): SOC through VAS format Sample 2 (*n* = 22): SOC and SMI through VAS format	Sweden	Sample 1: 41 Sample 2: 43	--	SOC-29	Sample 1: 152 (17) Sample 2: 143 (17)	Cross-sectional study	Self-Motivation Inventory (SMI)	No differences in SOC scores means between the participating groups, who had questionnaire administered through different formats. Negative correlation between SOC and SMI (r = −0,685, p < 0.001)
Lewis 1991	238 nurses 224 females (94%) 14 males (6%)	Nurses working in dialysis units	USA	36.1	--	SOC-29	143.1	Cross-sectional study	Nursing stress scale Maslach Burnout inventory (MBI)	Men show lower SOC scores than women Inverse correlation between SOC and overall stress (r = −0.39) Correlation between SOC and burnout subscales (emotional exhaustion r = −0.57, depersonalization r = −0.54, personal accomplishment r = 0.53) No relationship between SOC and age, marital status, educational level, position, years in nursing, number of patients or shift unit, shift length or hours worked per week.
